# Caring for Health Professionals in the COVID-19 Pandemic Emergency: Toward an “Epidemic of Empathy” in Healthcare

**DOI:** 10.3389/fpsyg.2020.01431

**Published:** 2020-06-09

**Authors:** Serena Barello, Guendalina Graffigna

**Affiliations:** EngageMinds HUB, Consumer, Food & Health Engagement Research Center, Department of Psychology, Università Cattolica del Sacro Cuore, Milan, Italy

**Keywords:** COVID-19, medical humanities, empathy, healthcare professionals, patient-doctor communication, medical psychology

## Abstract

Psychological research into healthcare opened the door to understanding people's emotional reactions when experiencing events perceived as life-threatening. This is the case of the current outbreak of the novel Coronavirus disease (COVID-19) that has recently been declared “a public health emergency of international concern (PHEIC)” by the World Health Organization (WHO). The response to an influenza pandemic might generate remarkable stress and emotional turmoil to healthcare providers who work during the outbreak. Prior experience with disasters, pandemics, and major traumatic events indicates that enhanced support to healthcare professionals enabling them to become aware of their own emotions and effectively share their perspective and lived experience with patients can help them in remaining efficient and focused during these stressful events. This outbreak marks a vital moment where healthcare systems can endorse an “epidemic of empathy” aimed at bringing science and humanism together to benefit patients and consolidate citizens' trust in healthcare providers during this and future healthcare crisis. Perhaps, the greatest opportunity for managing people fears during health emergencies—like the COVID-19 one—lies, in the short term, in restoring our connections with each other. Today, we are all called to rebuild a sense of community and the ties that bind us together as human beings.

Psychological research into healthcare opened the door to understanding people's emotional reactions when experiencing events perceived as life-threatening. This is the case of the current outbreak of the novel Coronavirus disease (COVID-19) that has been recently declared “a public health emergency of international concern (PHEIC)” by the World Health Organization (Wang et al., 2020).

When coping with a large-scale emergency like this, people often report a wide range of psychological needs, including out-of-control emotional reactions, as demonstrated by recent studies on the psychological impact of COVID-19 on populations across countries (Leon, 2004; Graffigna et al., 2020; Li et al., 2020; Lima et al., 2020). This “emotional surge” has the potential to overwhelm the medical system for as long as the public health crisis lasts. People's emotions, however, are only half of the story in a healthcare crisis.

The response to an influenza pandemic—like the one we are currently experiencing—might generate remarkable stress and emotional turmoil in healthcare providers who work during the outbreak (Maunder et al., 2008; Barello et al., 2020; Lancet, 2020). This issue has been shown by many studies on healthcare professionals' experience when facing the COVID-19 pandemic to be one that needs to be urgently addressed (Adams and Walls, 2020; Selman et al., 2020; Williamson et al., 2020). In these circumstances, health professionals become increasingly crucial points of reference for citizens regarding information on how to cope with the health crisis. This might make them feel fully responsible for managing the situation and often impede their ability to recognize their own human feelings, worries, and concerns (Khalid et al., 2016).

The expression of emotions by healthcare providers has been traditionally considered unprofessional and inconvenient, basically a sort of “taboo” (Meier et al., 2001). Research in this field has increasingly addressed this issue. This as a result of the fact that healthcare providers often have to deal with unexpected emotions arising from both the patient and themselves, and should find strategies to manage the stresses and anxieties of confronting illness and suffering (Meier et al., 2001; Delfrate et al., 2018). Indeed—although medical education does not explicitly promote healthcare workers “alexithymia” and emotional neglect – what frequently occurs in the practice with patients, especially through the action of the so called “hidden curriculum” (Cherry et al., 2014), seems to encourage clinicians to detach themselves from emotions (Shapiro, 2011b). Accordingly, clinician's socialization and professional implicit norms often foster health providers' emotional detachment (Halpern, 2001) as a strategy to cope with emotional challenges in interactions with patients (Rosenfield and Jones, 2004). At the same time, research has established that emotional regulation and disclosure among healthcare professionals may vary by cultural context (Rakovski and Price-Glynn, 2010; Mastracci and Hsieh, 2016). Moreover, studies on professionals' emotions highlight the importance of clinicians' awareness of their emotional states during the clinical relationship with their patients (Kushnir et al., 2011), although with some differences across clinical settings which have been supported by various practices in this regard (Halpern, 2014).

We know that emotions play a significant role in human interactions, even those occurring in healthcare encounters; as a matter of fact, they are a “vehicle” that is able not only to communicate intentions and shape behaviors, but that is also functional to build (or not) mutual trust, affect information processing, and even to determine people's health choices (Chapman and Coups, 2006). Studies showed that unrecognized emotions in the healthcare providers' experience may prevent the adoption of a patient-centered style of care and may be associated with harmful behaviors, such as neglecting patients' psychological issues or avoiding bonding with patients to elude the burden of highly emotional contents (Ely et al., 1995; Smith et al., 2005). Lack of recognition of emotions (of both patients and providers) can affect the quality of medical care and the healthcare provider's own sense of well-being, and may also lead to physician distress, disengagement, and burnout (Ekman and Halpern, 2015; Silva and Carvalho, 2016).

Prior experience with disasters, pandemics, and major traumatic events indicates that enhanced support to healthcare professionals enabling them to elaborate upon and become aware of their own emotions and effectively share their perspective and lived experience with patients can help them in remaining efficient and focused during these stressful events (Silva and Carvalho, 2016). That's because healthcare is not simply a purely scientific discipline, it is a matter of empathy, and communication skills are necessary to convey that empathy (Reynolds and Quinn Crouse, 2008).

During a healthcare crisis, an empathetic style of communication is the most effective when attempting to push the population to take preventive actions or to avoid harmful behaviors. An empathetic response, and the relative efforts in responding sensitively to others, has been associated with a more frequent adoption of recommended health precautions during a pandemic (Novack et al., 1997; King et al., 2016).

In fact, empathy, that involves commitment to understanding what others are feeling by adopting their perspective and responding in supportive ways, has been associated with benefits not only for laypeople but also for health providers. Sharing emotions, concerns, and worries by both could make all the actors involved in a healthcare crisis feel more responsible and aware of how much everyone's contribution could be determinant in effectively coping with the stressful consequences of such an event (King et al., 2016). Empathy has also been demonstrated to be a core element of an effective therapeutic relationship and to be a protective factor for health professionals emotional exhaustion (Wilkinson et al., 2017). On the other hand, studies have shown how, despite being an important component in providing effective care, empathy also generates vulnerability for stress-related symptoms such as compassion fatigue and professional emotional exhaustion and burnout (Hensley, 2008). The cognitive and emotional effort involved in empathic responses might strain the already overwhelmed psychological resource clinicians have in periods of high stress—like the COVID-19 emergency—, contributing to burnout and even causing emotional pain (Gleichgerrcht and Decety, 2013). These contradictory effects of empathy can be explained by considering that empathy is by nature multidimensional, interpersonal, and shaped by context and settings (Lamothe et al., 2014). According to Davis (2018), a core component of empathy in the context of patient care is perspective taking. It consists of adopting the point of view of another person and seeing things from their perspective. Perspective taking has been demonstrated to increase patient satisfaction (Blatt et al., 2010), as well as physician's well-being (Shanafelt et al., 2005). Empathetic concern, on the other hand, which is conceptually closer to sympathy, is the emotional reaction of an individual who is attentive to others' experience and spontaneously engages in helping behaviors (Lebowitz and Dovidio, 2015). It is important to distinguish the two concepts because they may lead to different outcomes. While perspective taking has been viewed to be always beneficial in patient care, a too elevated level of empathic concern could interfere with objectivity in diagnosis and treatment (Gleichgerrcht and Decety, 2013). Therefore, some effective detachment between clinicians and their patients has been considered desirable to maintain both clinical neutrality and emotional balance (Hojat et al., 2003). Moreover, other dimensions such as personal authenticity and hope do interact with empathy-related processes and outcomes and should be considered as other aspects to be trained in medical education programs (Shapiro, 2011a; Ünal, 2014; Yagil and Shnapper-Cohen, 2016).

Only when health professionals and citizens opt for a relationship where emotional disclosures about events could occur, could their interaction become a true partnership with shared decision-making authority and mutual responsibility for outcomes, thus reducing stress and frustration from both sides. To gain this objective, health systems are warranted to recognize that healthcare professionals are humans too by legitimizing their empathetic response; however, a practical plan to strengthen the healthcare providers psychological resilience and work engagement during pandemic emergencies is needed to prevent them from becoming “second victims” in this scenario (Scott et al., 2009) and to experience the “side effects” related to empathy. In other words, during health emergencies, like the one that we are currently experiencing with COVID-19, health professionals should be emotionally supported and safeguarded from the risk of forgetting their human side. If not, the consequences of the pandemic has to also take into account the psychological costs related to the increasing burnout rates among the health workforce.

This outbreak marks a vital moment where healthcare systems could begin to endorse an “epidemic of empathy” aimed at bringing science and humanism together to benefit patients and consolidate citizens' trust in healthcare providers during a healthcare crisis. Perhaps the greatest opportunity for managing people's fears during health emergencies—like the COVID-19 one—lies, in the short term, in restoring our connections with each other. Today, we are all called to rebuild a sense of community and the ties that bind us together as human beings.

## Data Availability Statement

The original contributions presented in the study are included in the article/supplementary material, further inquiries can be directed to the corresponding author/s.

## Author Contributions

SB drafted and edited the manuscript. GG critically revised the manuscript. Both authors approved the contributions for publication.

## Conflict of Interest

The authors declare that the research was conducted in the absence of any commercial or financial relationships that could be construed as a potential conflict of interest.

## References

[B1] Adams J. G. Walls R. M. (2020). Supporting the health care workforce during the COVID-19 global epidemic. JAMA 323, 1439–1440. 10.1001/jama.2020.3972 32163102

[B2] Barello S. Palamenghai L. Graffigna G. (2020). Burnout and somatic symptoms among frontline healthcare professionals at the peak of the Italian COVID-19 Pandemic. Psychiatry Res. 290:113129. 10.1016/j.psychres.2020.113129 32485487 PMC7255285

[B3] Blatt B. LeLacheur S. F. Galinsky A. D. Simmens S. J. Greenberg L. (2010). Does perspective-taking increase patient satisfaction in medical encounters? Acad. Med. 85, 1445–1452. 10.1097/ACM.0b013e3181eae5ec 20736672

[B4] Chapman G. B. Coups E. J. (2006). Emotions and preventive health behavior: worry, regret, and influenza vaccination. Health Psychol. 25:82. 10.1037/0278-6133.25.1.82 16448301

[B5] Cherry M. G. Fletcher I. O'Sullivan H. Dornan T. (2014). Emotional intelligence in medical education: a critical review. Med. Educ. 48, 468–478. 10.1111/medu.12406 24712932

[B6] Davis M. H. (2018). Empathy: A Social Psychological Approach. New York, NY: Routledge 10.4324/9780429493898

[B7] Delfrate F. Ferrara P. Spotti D. Terzoni S. Lamiani G. Canciani E.. (2018). Moral Distress (MD) and burnout in mental health nurses: a multicenter survey. Med. Lav 109, 97–109. 10.23749/mdl.v109i2.6876 29701626 PMC7682177

[B8] Ekman E. Halpern J. (2015). Professional distress and meaning in health care: why professional empathy can help. Soc. Work Health Care 54, 633–650. 10.1080/00981389.2015.1046575 26317765

[B9] Ely J. W. Levinson W. Elder N. C. Mainous A. G. Vinson D. C. (1995). Perceived causes of family physicians' errors. J. Family Practice 40, 337–344. 7699346

[B10] Gleichgerrcht E. Decety J. (2013). Empathy in clinical practice: how individual dispositions, gender, and experience moderate empathic concern, burnout, and emotional distress in physicians. PLoS ONE 8:e61526. 10.1371/journal.pone.0061526 23620760 PMC3631218

[B11] Graffigna G. Barello S. Savarese M. Palamenghi L. Castellini G. Bonanomi A. (2020). Measuring Italian Citizens′ engagement in the first wave of the COVID-19 pandemic containment measures a cross-sectional study. medRxiv [preprint]. 10.1101/2020.04.22.20075234 PMC7485890 32915822

[B12] Halpern J. (2001). From Detached Concern to Empathy: Humanizing Medical Practice. New York, NY: Oxford University Press 10.1093/acprof:osobl/9780195111194.001.0001

[B13] Halpern J. (2014). From idealized clinical empathy to empathic communication in medical care. Med. Health Care Philosop. 17, 301–311. 10.1007/s11019-013-9510-4 24343367

[B14] Hensley P. L. (2008). Help for the helper: the psychophysiology of compassion fatigue and vicarious trauma. Bull. Menninger Clin 72:79.

[B15] Hojat M. Gonnella J. S. Mangione S. Nasca T. J. Magee M. (eds.). (2003). Physician empathy in medical education and practice: experience with the Jefferson Scale of Physician Empathy, in Seminars in Integrative Medicine, Vol. 1 (Philadelphia, PA: WB Saunders), 25–41. 10.1016/S1543-1150(03)00002-4

[B16] Khalid I. Khalid T. J. Qabajah M. R. Barnard A. G. Qushmaq I. A. (2016). Healthcare workers emotions, perceived stressors and coping strategies during a MERS-CoV outbreak. Clin. Med. Res. 14:7–14. 10.3121/cmr.2016.1303 26847480 PMC4851451

[B17] King D. B. Kamble S. DeLongis A. Sandman P. M. Lanard J. Silva J. V. (2016). Coping with influenza A/H1N1 in India: empathy is associated with increased vaccination and health precautions. EMA 26, 31–37. 10.1080/14635240.2016.1174950

[B18] Kushnir T. Kushnir J. Sarel A. Cohen A. H. (2011). Exploring physician perceptions of the impact of emotions on behaviour during interactions with patients. Fam. Pract. 28, 75–81. 10.1093/fampra/cmq070 20833703

[B19] Lamothe M. Boujut E. Zenasni F. Sultan S. (2014). To be or not to be empathic: the combined role of empathic concern and perspective taking in understanding burnout in general practice. BMC Fam. Pract. 15, 1–7. 10.1186/1471-2296-15-15 24456299 PMC3914722

[B20] Lancet T. (2020). COVID-19: protecting health-care workers. Lancet 395:922. 10.1016/S0140-6736(20)30644-9 32199474 PMC7138074

[B21] Lebowitz M. S. Dovidio J. F. (2015). Implications of emotion regulation strategies for empathic concern, social attitudes, and helping behavior. Emotion 15, 187–194. 10.1037/a0038820 25706828

[B22] Leon G. R. (2004). Overview of the psychosocial impact of disasters. Prehosp. Disaster Med. 19, 4–9. 10.1017/S1049023X00001424 15453154

[B23] Li S. Wang Y. Xue J. Zhao N. Zhu T. (2020). The impact of COVID-19 epidemic declaration on psychological consequences: a study on active Weibo users. Int. J. Environ. Res. Public Health 17:2032. 10.3390/ijerph17062032 32204411 PMC7143846

[B24] Lima C. K. T. de Medeiros Carvalho P. M. Lima I. D. A. S. de Oliveira Nunes J. V. A. Saraiva J. S. de Souza R. I.. (2020). The emotional impact of Coronavirus 2019-nCoV (new Coronavirus disease). Psychiatry Res. 287:112915. 10.1016/j.psychres.2020.112915 32199182 PMC7195292

[B25] Mastracci S. Hsieh C. W. (2016). Emotional labor and job stress in caring professions: exploring universalism and particularism in construct and culture. Int. J. Public Adm. 39, 1125–1133. 10.1080/01900692.2015.1068327

[B26] Maunder R. G. Leszcz M. Savage D. Adam M. A. Peladeau N. Romano D.. (2008). Applying the lessons of SARS to pandemic influenza. Can. J. Public Health 99, 486–488. 10.1007/BF03403782 19149392 PMC5148615

[B27] Meier D. E. Back A. L. Morrison R. S. (2001). The inner life of physicians and care of the seriously ill. JAMA 286, 3007–3014. 10.1001/jama.286.23.3007 11743845

[B28] Novack D. H. Suchman A. L. Clark W. Epstein R. M. Najberg E. Kaplan C. (1997). Calibrating the physician: personal awareness and effective patient care. JAMA 278, 502–509. 10.1001/jama.1997.03550060078040 9256226

[B29] Rakovski C. C. Price-Glynn K. (2010). Caring labour, intersectionality and worker satisfaction: an analysis of the National Nursing Assistant Study (NNAS). Sociol. Health Illn 32, 400–414. 10.1111/j.1467-9566.2009.01204.x 19891615

[B30] Reynolds B. Quinn Crouse S. (2008). Effective communication during an influenza pandemic: the value of using a crisis and emergency risk communication framework. Health Promot. Pract. 9 (4 Suppl):13–17. 10.1177/1524839908325267 18936255

[B31] Rosenfield P. J. Jones L. (2004). Striking a balance: training medical students to provide empathetic care. Med. Educ. 38, 927–933. 10.1111/j.1365-2929.2004.01931.x 15327673

[B32] Scott S. D. Hirschinger L. E. Cox K. R. McCoig M. Brandt J. Hall L. W. (2009). The natural history of recovery for the healthcare provider “second victim” after adverse patient events. BMJ Qual. Saf. 18, 325–330. 10.1136/qshc.2009.032870 19812092

[B33] Selman L. E. Chao D. Sowden R. Marshall S. Chamberlain C. Koffman J. (2020). Bereavement support on the frontline of COVID-19: recommendations for hospital clinicians. J. Pain Symptom Manage. 10.1016/j.jpainsymman.2020.04.024. [Epub ahead of print]. 32376262 PMC7196538

[B34] Shanafelt T. D. West C. Zhao X. Novotny P. Kolars J. Habermann T.. (2005). Relationship between increased personal well-being and enhanced empathy among. J. Gen. Intern. Med. 20, 559–564. 10.1007/s11606-005-0102-8 16050855 PMC1490167

[B35] Shapiro J. (2011a). Illness narratives: reliability, authenticity and the empathic witness. Med. Humanit 37:68–72. 10.1136/jmh.2011.007328 21757469

[B36] Shapiro J. (2011b). Perspective: does medical education promote professional alexithymia? A call for attending to the emotions of patients and self in medical training. Acad. Med. 86, 326–332. 10.1097/ACM.0b013e3182088833 21248595

[B37] Silva J. V. Carvalho I. (2016). Physicians experiencing intense emotions while seeing their patients: what happens? Perm. J. 20, 31–37. 10.7812/TPP/15-229 27479947 PMC4991912

[B38] Smith R. C. Dwamena F. C. Fortin A. H. (2005). Teaching personal awareness. J. Gen. Intern. Med. 20, 201–207. 10.1111/j.1525-1497.2005.40212.x 15836555 PMC1490050

[B39] Ünal Z. (2014). The contribution of emotional intelligence on the components of burnout: the case of health care sector professionals. Electr. J. Business Ethics Organ. Studies 19, 27–34.

[B40] Wang C. Pan R. Wan X. Tan Y. Xu L. Ho C. S.. (2020). Immediate psychological responses and associated factors during the initial stage of the 2019 coronavirus disease (COVID-19) epidemic among the general population in China. Int. J. Environ. Res. Public Health 17:1729. 10.3390/ijerph17051729 32155789 PMC7084952

[B41] Wilkinson H. Whittington R. Perry L. Eames C. (2017). Examining the relationship between burnout and empathy in healthcare professionals: a systematic review. Burnout Res. 6, 18–29. 10.1016/j.burn.2017.06.003 28868237 PMC5534210

[B42] Williamson V. Murphy D. Greenberg N. (2020). COVID-19 and experiences of moral injury in front-line key workers. Occup. Med. 10.1093/occmed/kqaa052. [Epub ahead of print]. 32239155 PMC7184422

[B43] Yagil D. Shnapper-Cohen M. (2016). When authenticity matters most: physicians' regulation of emotional display and patient satisfaction. Patient Educ. Couns. 99, 1694–1698. 10.1016/j.pec.2016.04.003 27085519

